# Identification of a novel *RPGR* mutation associated with retinitis pigmentosa and primary ciliary dyskinesia in a Slovak family: a case report

**DOI:** 10.3389/fped.2024.1339664

**Published:** 2024-01-25

**Authors:** Zuzana Kolkova, Peter Durdik, Veronika Holubekova, Anna Durdikova, Milos Jesenak, Peter Banovcin

**Affiliations:** ^1^ Biomedical Center Martin, Jessenius Faculty of Medicine in Martin, Comenius University in Bratislava, Martin, Slovakia; ^2^ Department of Pediatrics, Jessenius Faculty of Medicine in Martin, Comenius University in Bratislava, Martin, Slovakia; ^3^ Department of Pediatrics, University Hospital Martin, Martin, Slovakia; ^4^ Department of Pulmonology and Phthisiology, Jessenius Faculty of Medicine in Martin, Comenius University in Bratislava, University Hospital in Martin, Martin, Slovakia; ^5^ Department of Clinical Immunology and Allergology, University Hospital in Martin, Martin, Slovakia

**Keywords:** RPGR gene, mutation, retinitis pigmentosa, primary ciliary dyskinesia, X-linked inheritance

## Abstract

**Background:**

The mutations in the *RPGR* (retinitis pigmentosa GTPase regulator) gene are the most common cause of X-linked retinitis pigmentosa (XLRP), a rare genetic disorder affecting the photoreceptor cells in the retina. Several reported cases identified this gene as a genetic link between retinitis pigmentosa (RP) and primary ciliary dyskinesia (PCD), characterised by impaired ciliary function predominantly in the respiratory tract. Since different mutations in the same gene can result in various clinical manifestations, it is important to describe a correlation between the gene variant and the observed phenotype.

**Methods:**

Two young brothers from a non-consanguineous Slovak family with diagnosed retinal dystrophy and recurrent respiratory infections were examined. Suspected PCD was diagnosed based on a PICADAR questionnaire, nasal nitric oxide analysis, transmission electron microscopy, high-speed video microscopy analysis, and genetic testing.

**Results:**

We identified a novel frameshift *RPGR* mutation NM_001034853: c.309_310insA, p.Glu104Argfs*12, resulting in a complex X-linked phenotype combining PCD and RP. In our patients, this mutation was associated with normal ultrastructure of respiratory cilia, reduced ciliary epithelium, more aciliary respiratory epithelium, shorter cilia, and uncoordinated beating with a frequency at a lower limit of normal beating, explaining the clinical manifestation of PCD in our patients.

**Conclusion:**

The identified novel pathogenic mutation in the *RPGR* gene expands the spectrum of genetic variants associated with the X-linked PCD phenotype overlapping with RP, highlighting the diversity of mutations contributing to the disorder. The described genotype–phenotype correlation can be useful in clinical practice to recognise a broader spectrum of PCD phenotypes as well as for future research focused on the genetic basis of PCD, gene interactions, the pathways implicated in PCD pathogenesis, and the role of RPGR protein for the proper functioning of cilia in various tissues throughout the body.

## 1 Introduction

Primary ciliary dyskinesia (PCD) is a rare, clinically and genetically heterogeneous multisystem disorder characterised by recurrent respiratory infections, chronic inflammation, bronchiectasis, sinusitis, and impaired fertility. The cause of the respiratory conditions is ineffective mucociliary clearance resulting from defects in the structure, assembly, and function of motile cilia as a result of pathogenic mutations in more than 50 PCD relevant genes described up to now (1). Since the structure of motile and nodal cilia is similar, gene mutations negatively affect the motility and function of nodal cilia during embryogenesis, leading to laterality defects associated with PCD (2). PCD is typically inherited in an autosomal recessive manner, but rare cases of X-linked inheritance have also been described, especially in a syndromic phenotype linked to retinitis pigmentosa (3).

Retinitis pigmentosa (RP) is a hereditary disease characterised by progressive retinal degeneration caused by cell death of photoreceptors. Patients suffer from slowly progressing night blindness, loss of peripheral vision, and even loss of central vision. RP can be inherited in an autosomal dominant, recessive, X-linked, and even mitochondrial pattern (4). More than 70% of X-linked RP (XLRP) and about 10%–15% of all RP cases are caused by mutations in the *RPGR* gene that encodes the retinitis pigmentosa GTPase regulator located within the connecting cilium between the inner and outer segments of the human rod and cone cells (5, 6). The function of the RPGR protein is not completely clear, but it is proposed that, together with its interacting proteins, it plays an important role in the regulation of protein transport across the connecting cilium, and mutations causing its dysfunction can lead to photoreceptor cell death (7, 8). In addition to the retina, RPGR isoforms resulting from alternative transcription and post-translational modifications were also detected in other tissues, such as the lung, kidney, and brain (9–11). Based on the role of the RPGR protein in intraflagellar transport, it can be assumed that mutations negatively affect the assembly and viability of motile and immotile cilia. Several cases of XLRP associated with recurrent respiratory infections and impaired hearing have been observed (3, 12–18).

Here, we describe a case of two brothers with complex X-linked clinical phenotypes combining PCD with RP, two rare genetic disorders linked by a novel hemizygous frameshift mutation in the *RPGR* gene. This case report contributes to the suggestion that RPGR protein plays an essential role in ciliary function in the retina and other tissues. Understanding the relationship between *RPGR* mutations and PCD is important for improving diagnosis, treatment, and genetic counselling for families affected by these conditions.

## 2 Case report

### 2.1 Case presentation

We examined two brothers, a 13-year-old (P1) and a 10-year-old (P2), from a non-consanguineous Slovak family in the national PCD centre. They were recruited to identify suspicious PCD due to retinal dystrophy, recurrent mostly upper respiratory infections, almost recurrent rhinosinusitis, and bronchiectasis of unknown origin on computed tomography (CT) examination in the case of the older boy. The routine clinical examinations excluded the usual causes of recurrent respiratory infections, including developmental congenital defects, allergic diseases, primary immunodeficiency, gastroesophageal reflux disease, and otorhinolaryngological causes (polyps, adenoid vegetation). Cystic fibrosis was excluded by negative newborn screening provided as standard in our country (the determination of immunoreactive trypsinogen in a dry drop of blood) and by demonstration of normal sweat tests. The PCD diagnostic algorithm was based on the ERS (European Respiratory Society) guidelines (19).

#### 2.1.1 Clinical features

Both boys were term-birth babies with no neonatal respiratory distress and no neonatal rhinitis. There were no congenital heart defects, hydrocephalus, or laterality defects. A history of multi-trigger persistent wheezing, recurrent respiratory tract infections, persistent nasal congestion, and chronic rhinosinusitis was presented mostly from 1 year of life and worsened after joining the collective in both boys. The initial retinal degenerative symptoms started in the older boy (P1) at the age of 5 years with decreased night vision following the worsening of the mid-peripheral visual field. The retinal degeneration with visual impairment, RP, was diagnosed at the age of 8 years by a combination of visual field tests, visual acuity tests, and fluorescein angiography. Approximately 1 year later, the bronchiectasis localised in the right middle lobe was confirmed by a high-resolution CT examination. At the age of 10 years, the older boy was sent to the national PCD centre. Neurological examination, hearing, and lung functions were normal. In the younger brother (P2), deterioration of night vision occurred at the age of 6 years and was confirmed in the same way as in the older brother at 7 years. He was sent to the national PCD centre 1 year later.

### 2.2 Diagnosis of PCD

#### 2.2.1 PICADAR questionnaire

Patients were interviewed and scored for probable PCD using the PICADAR questionnaire (20). The PICADAR level for both patients was three points (two points for term-baby and one point for chronic rhinosinusitis), which is not significant for PCD patients.

#### 2.2.2 Nasal nitric oxide analysis

For nasal nitric oxide (nNO), an electrochemical analyser NIOX VERO (Circassia AB, Uppsala, Sweden) was used. The measurement was performed under standard conditions in the laboratory. The sampling rate was 0.3 nL/min, and the ambient nitric oxide (NO) was 6 ppb. The results were also confirmed by the breath-hold manoeuvre, and a difference of less than 10% was considered normal. The examination was graded C depending on the ERS technical standard (21). The nNO was 249 ppb (74.7 nL/min) on the left nostril and 261 ppb (78.3 nL/min) on the right nostril in the older patient (P1) and 234 ppb (70.2 nL/min) on the left nostril and 252 ppb (75.6 nL/min) on the right nostril in the younger patient (P2). The measured values were at the limit of significance for PCD.

#### 2.2.3 Collection of respiratory epithelial cells and high-speed video microscopy analysis

After 3 weeks of no infection or no relapse of chronic symptoms, ciliary cells were obtained by brushing the inferior turbinate using a cytology brush (CytobrushCYTST Changzhou Shunfeng Plastic Co., LM, China). The samples of ciliated epithelium were suspended in RPMI 1640 Medium warmed up to 35°C. The ciliary beat was measured using a digital high-speed imaging workstation, designed by a team of researchers from Jessenius Medical Faculty in Martin and the University of Žilina (both in Slovakia), for the high-speed video microscopy analysis (HSVMA). A detailed description of this method (Utility Model Number 6811) (22) has been published in several papers (23–25). The warmed-up samples were evaluated under an inverted phase-contrast microscope (Zeiss, Oberkochen, Germany) at × 400 magnification, and ciliary movement was recorded with a digital high-speed video (Basler A504KC; Basler AG, Germany) with a frame rate of 500 Hz. Each microscope slide was examined within the first 10 min after its preparation, and more than 10 sequences with the best ciliary kinematics were recorded. The video sequences were played back frame by frame using the software application Ciliary Analysis (National Instruments LabVIEW development environment). The cilia beat frequency (CBF) was determined by calculating the mean of all recorded cilia beat cycles, and the cilia beating pattern (CBP) was evaluated on slow-motion playbacks (up to 10 times slower) and determined by two independent expert operators. The range of mean ciliary beat frequency was 5.5–7.5 Hz in both patients, which represented the lower limit of the normal ciliary frequency. There was a higher percentage of aciliary respiratory epithelium, and most of the cilia were shorter (Supplementary Video S1). The ciliary beat pattern analysis revealed a strong increase in ciliary bundles beating in an uncoordinated manner (Supplementary Video S2).

#### 2.2.4 Transmission electron microscopy

**Figure 1:**
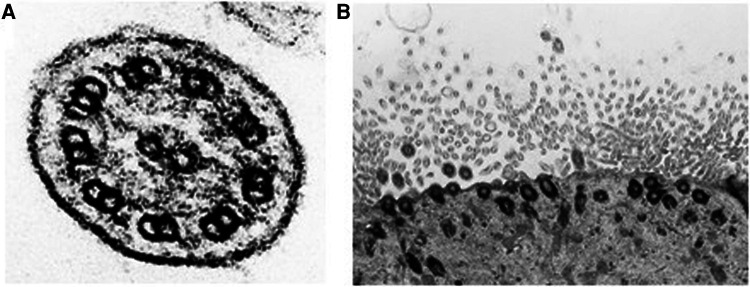
(**A**) Cross section of respiratory cilia. No structural abnormalities were observed by TEM; normal 9 + 2 configuration of microtubules and the presence of outer and inner arms are shown. (**B**) Reduced ciliary epithelium and shorter cilia are described.

Respiratory epithelial cells from bronchoscopy or brushing from the middle nasal turbinate were collected and fixed in 3% glutaraldehyde. The transmission electron microscopy (TEM) analysis was performed by certified laboratory CYTOPATHOS, the centre for ciliary ultrastructure analysis. Our patients did not reveal ciliary ultrastructure abnormalities, with no changes of class 1 (Hallmark diagnostic defects) and class 2 defects for the ultrastructural diagnosis (Figure 1A). Reduced ciliary epithelium, more aciliary respiratory epithelium, and shorter cilia were described (Figure 1B).

### 2.3 Mutational analysis

**Figure 2:**
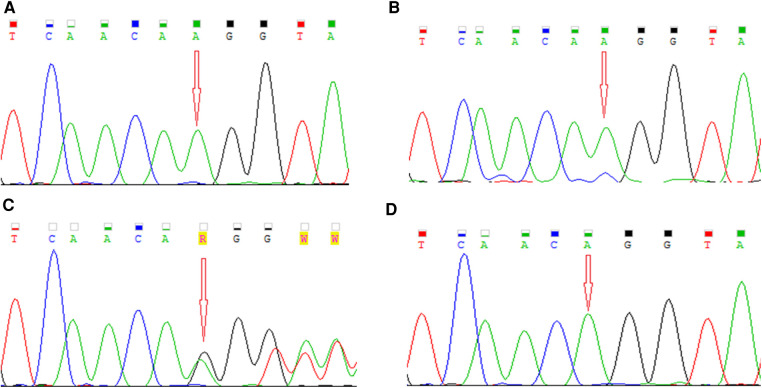
Electropherograms showing detected mutation NM_001034853: c.309_310insA, p.Glu104Argfs*12 in the *RPGR* gene in (**A, B**) a hemizygous state in our patients, (**C**) in a heterozygous state in their mother, and (**D**) the wild-type allele in their father.

DNA was isolated from peripheral venous blood samples of both patients and their parents. In the older boy (P1), genetic analysis of coding and splicing-relevant regions of 35 genes associated with PCD was performed by the targeted next generation sequencing (NGS) method using hybridisation solution Nextera Flex Pre-enrichment and TruSight One expanded panel (Illumina, USA) on NextSeq 550 (Illumina, USA). Illumina bcl2fastq v2.20 and SEQNEXT v5.2.0 (JSI medical systems) software were used for bioinformatical analysis with reference genome *Homo sapiens* GRCh37/hg19 and the RefSeq database. NGS analysis detected one nucleotide insertion in exon 4 of the *RPGR* gene in the hemizygous state, NM_001034853: c.309_310insA, p.Glu104Argfs*12. Sanger sequencing was used to verify the detected variant in the patient and his younger brother, as well as for segregation analysis in the family. The sequences of primers designed to intron regions were as follows: forward 5′-GTCCTGGACTACTGTTCATTTT-3′, reverse 5′-AAGCCACGTTACTGGAATGAG-3′. Sequencing analysis was performed on the ABI3500 Genetic Analyzer (Applied Biosystems, USA). The pathogenicity and functional effect of the identified variant were assessed by the *in silico* predictive software MutationTaster (26). The detected variant was confirmed by Sanger sequencing in the older patient (P1) as well as in his younger brother (P2) (Figures 2A, B). The segregation analysis confirmed the maternal origin of the mutation (Figure 2C). As expected, the father has a wild-type genotype (Figure 2D). The identified *RPGR* mutation NM_001034853: c.309_310insA, p.Glu104Argfs*12 located at the end of exon 4 was not found in databases ExAC, 1000G, or ClinVar and to the best of our knowledge, no report with this variant has been published so far. The consequence of the insertion of nucleotide A within the coding sequence is a shift in the reading frame and the creation of a premature stop codon. Based on the prediction in the MutationTaster program, premature termination of translation leads to nonsense-mediated decay, and this variant is predicted to be disease-causing. According to the ACMG (American College of Medical Genetics and Genomics) guidelines, this variant is classified as pathogenic (PVS1 and PM2 category) (27).

### 2.4 Family history

**Figure 3:**
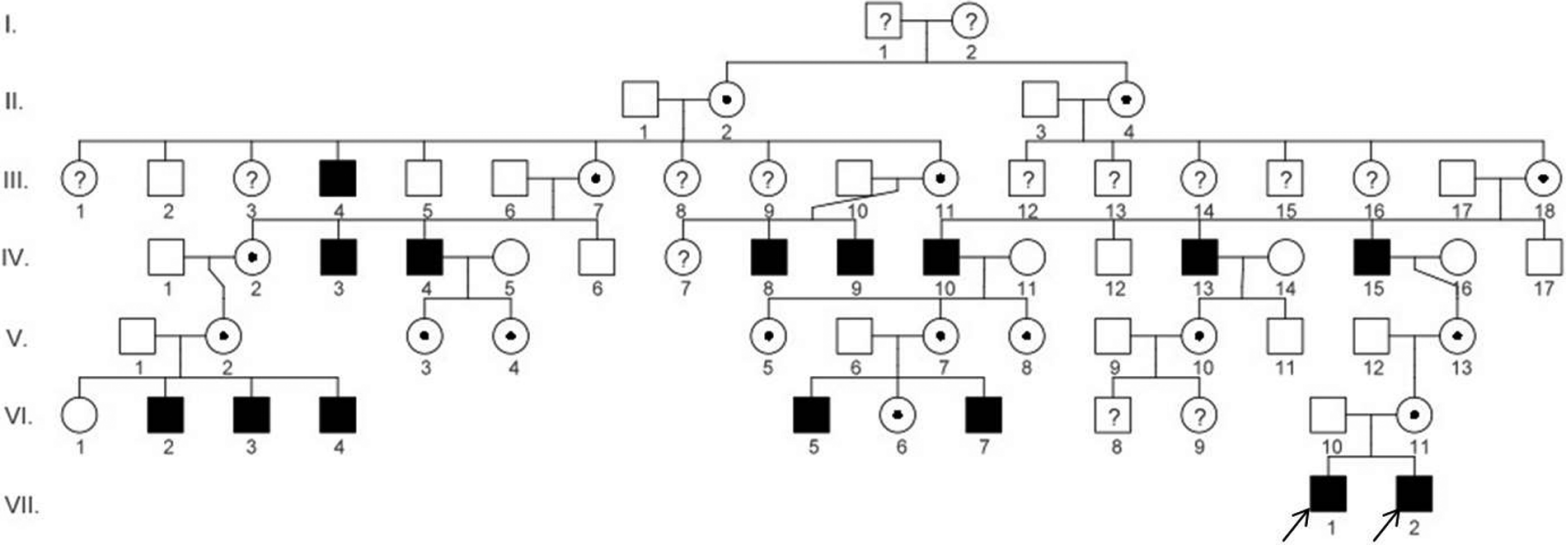
Pedigree diagram of the family showing disease segregation. The arrows indicate the patients (P1 and P2) described in this case report. The pedigree chart was created in the program QuickPed (28).

Due to X-linked inheritance, we also analysed other members of the boys' family for the presence of typical respiratory symptoms or visual disturbances typical for RP. On the basis of medical data, a slight visual impairment, myopia, was detected in the boys' mother and grandmother without respiratory symptoms. Subsequently, we searched the complete Slovak PCD database with a focus on the connection between RP and PCD and identified five more boys who had an identical mutation in the *RPGR* gene (c.309_310insA) and similar clinical features. Based on the family tree provided by the family, distant kinship relationships between patients were clarified. The six-generation pedigree chart is shown in Figure 3. In the diagram, the two patients described above are marked as VII.1 (P1) and VII.2 (P2). The family members VI.2, VI.3, VI.4, VI.5, and VI.7 were also examined in the Slovak PCD centre, owing to the presence of RP and PCD symptoms, by the methods described above. The complete clinical features and results of the structural and functional analysis of the respiratory cilia of all affected boys are summarised in Table 1. From the available medical records, it is known that affected men in the family (III.4, IV.3, IV.4, IV.8, IV.9, IV.10, IV.13, IV.10) suffered from night blindness, but the presence of PCD signs is unknown. In female carriers, varying degrees of myopia were present.

**Table 1:** Characteristics and results of diagnostic test for PCD in all examined patients.

No.	Gender	Age at diagnosis (years)	Clinical feature	PICADAR	nNO (left/right nostril in nL/min)	HSVM—ciliary frequency (Hz)	HSVM—ciliary pattern	TEM
VII.1 (P1)	Male	10	No perinatal history, no laterality defects, early onset of chronic rhinosinusitis, wet cough, bronchiectasis, retinitis pigmentosa	3/14	74.7/78.3	5.5–7.5	Uncoordinated movement, higher frequency of aciliary epithelium and microcilia	Normal ultrastructure of cilia, reduced ciliary epithelium, shorter cilia
VII.2 (P2)	Male	9	No perinatal history, no laterality defects, early onset of chronic rhinosinusitis, wet cough, retinitis pigmentosa	3/14	70.2/75.6	5.5–7.5	Uncoordinated movement, higher frequency of aciliary epithelium and microcilia	Normal ultrastructure of cilia, reduced ciliary epithelium, shorter cilia
VI.5	Male	14	No perinatal history, no laterality defects, early onset of chronic rhinosinusitis, recurrent otitis media and ventilation tubes, hearing impairment, wet cough, retinitis pigmentosa	4/14	56.7/51.3	5.0–8.0	Uncoordinated movement, higher frequency of aciliary epithelium and microcilia	Normal ultrastructure of cilia, reduced ciliary epithelium, shorter cilia
VI.7	Male	11	No perinatal history, no laterality defects, early onset of recurrent otitis media, chronic rhinosinusitis, retinitis pigmentosa	4/14	60.3/58.2	5.0–7.5	Uncoordinated movement, higher frequency of aciliary epithelium and microcilia	Normal ultrastructure of cilia, reduced ciliary epithelium, shorter cilia
VI.2	Male	15	No perinatal history, no laterality defects, early onset of recurrent rhinitis and otitis media with tubular tubes, hearing impairment, retinitis pigmentosa	4/14	54.0/50.7	5.5–7.5	Uncoordinated movement, higher frequency of aciliary epithelium	Normal ultrastructure of cilia, reduced ciliary epithelium
VI.3	Male	7	No perinatal history, no laterality defects, early onset of recurrent rhinitis and otitis media with tubular tubes, hearing impairment, lung atelectasis of right S4, retinitis pigmentosa	6/14	26.7/21.9	3.5–6.0	Uncoordinated movement, higher frequency of aciliary epithelium and microcilia	Normal ultrastructure of cilia, reduced ciliary epithelium, shorter cilia
VI.4	Male	5	No perinatal history, no laterality defects, recurrent rhinitis and otitis media, retinitis pigmentosa	3/14	18.6/19.5	4.5–7.0	Uncoordinated movement, higher frequency of aciliary epithelium and microcilia	Normal ultrastructure of cilia, reduced ciliary epithelium, shorter cilia

nNO, nasal nitric oxide; HSVM, high-speed video microscopy; TEM, transmission electron microscopy.

## 3 Discussion

Both RP and PCD are rare genetic diseases belonging to a very heterogeneous group of inherited defects in ciliary function called ciliopathies. RP, a progressive degenerative disorder of the retina, is caused by the loss of viability and cell death of photoreceptors that are modified primary cilia with sensory function. Conversely, PCD is a multisystem disorder of motile cilia characterised by impaired mucociliary clearance, leading to recurrent respiratory infections. Since motile cilia are also present outside the airways, patients often suffer from laterality abnormalities and subfertility. Since primary and motile cilia share structural features as well as many proteins necessary for their proper function, it is not surprising that mutations in some genes influence the function of both types of cilia, leading to syndromic manifestation with overlapping features of motile and non-motile ciliopathies. This is also the case of the *RPGR* gene associated with X-linked RP, but several cases of *RPGR* mutations leading to RP with respiratory symptoms have been published (3, 12, 14–17, 29, 30). This complex interplay between *RPGR* mutations, RP, and PCD underscores the importance of understanding the underlying molecular mechanisms and potential therapeutic approaches.

PCD is typically inherited in an autosomal recessive manner, but rare cases with autosomal dominant (31) or X-linked inheritance have also been described (3, 32, 33). We report the cases of two brothers with X-linked PCD associated with RP as a result of a novel frameshift mutation in the *RPGR* gene. Both patients were diagnosed with RP and were monitored because of the early onset of rhinosinusitis, recurrent respiratory infections, and bronchiectasis in the older brother. None of the boys suffered from neonatal respiratory distress, congenital heart defects, or laterality defects. For suspected PCD, they were examined for nNO measurement, ciliary ultrastructure, CBF, and PICADAR score. Lastly, genetic testing revealed mutation c.309_310insA (p.Glu104Argfs*12) located in exon 4 of the *RPGR* gene, which has not been found in databases or reported in the literature so far. The insertion of nucleotide A into the coding sequence results in a frameshift and premature termination of translation and subsequently presumably leads to nonsense-mediated decay. The character of this mutation was determined to be deleterious and disease-causing, which was also predicted by *in silico* analysis. Based on these diagnostic tests, we were able to confirm PCD diagnosis with X-linked inheritance.

The *RPGR* gene located on the X chromosome encodes more than 20 various isoforms resulting from alternative splicing and post-translational modifications that are tissue-specific (9, 10, 34). This explains the different levels of these isoforms in different tissues. There are two major widely expressed isoforms, *RPGR^1−19^* and *RPGR^ORF^*, that share exons 1–14 but differ in the C-terminus. In humans, the *RPGR^ORF^* isoform is expressed predominantly in the outer segment of photoreceptors in the retina and harbours the majority of mutations leading to XLRP (35). The constitutive *RPGR^1−19^* isoform is encoded by 19 exons and contains an isoprenylation signal in the C-terminus for its membrane trafficking and ciliary localisation (36–38). This isoform was detected, among others, in motile cilia of the respiratory tract, where it is localised in the transitional zone at the base of the cilium (10). The presence of defective RPGR protein in the respiratory cilia explains respiratory symptoms in RP patients with an *RPGR* mutation. The function of RPGR protein is not completely understood, but it is proposed that together with its interacting partners, it plays an important role in intraflagellar transport, ensuring protein trafficking in cilia, thereby maintaining their viability and proper function (39–41). Therefore, it can be assumed that mutations in the *RPGR* gene disrupt ciliary transport and signalling pathways, contributing to the development of both conditions. Nevertheless, it should be noted that not all *RPGR* mutations result in the PCD phenotype, and the clinical manifestation may vary between individuals.

All examined boys in the family had similar clinical manifestations of PCD: repeated rhinosinusitis and otitis, which started in early childhood in some of them. Eye symptoms such as decreased night vision and the worsening of the mid-peripheral visual field appeared at a young school age. In all patients, we observed normal ultrastructure of respiratory cilia, reduced or even aciliary epithelium, and shorter cilia. The HSVMA showed beat frequency at the lower limit of the normal range but an uncoordinated pattern of movement. McCray et al. reported normal ultrastructure of respiratory cilia but abnormal ciliary orientation associated with defects in the CBP in patients with XLRP without PCD symptoms (42). No abnormalities in ultrastructure were observed in PCD patients with *RPGR* mutations either, but in the case of the c.154G>A mutation, impaired ciliary orientation and uncoordinated beating of cilia were revealed (12). Normal ciliary beating and orientation are determined during ciliogenesis by the correct anchoring and orientation of the basal body in the plasma membrane, which is controlled through planar cell polarity (PCP) signalling (43, 44). It is suggested that RPGR protein is involved in cilia orientation by interacting with the components of the PCP pathway. Patnaik et al. demonstrated the role of RPGR and its interacting proteins (RPGRIP1 and RPGRIP2l) in actin cytoskeleton arrangement by regulating the stability of components in the PCP pathway (45). This can be one of the mechanisms contributing to the ciliopathies caused by *RPGR* mutations because the organisation of the actin cytoskeleton is important for proper ciliogenesis and ciliary orientation of the primary retinal cilia (40) as well as motile cilia (46).

The segregation analysis showed that the mother of our patients is a heterozygous carrier of the *RPGR* mutation consistent with X-linked inheritance. She suffers from myopia but has no respiratory symptoms, and PCD tests were negative, without abnormalities of HSVMA. The manifestation of retinal dystrophy was also recorded in the maternal grandmother as well as in other heterozygous carriers in the family. In the literature, a wide spectrum of visual dysfunction and abnormalities in the function and structure of photoreceptors were observed in female carriers, which is probably caused by mosaicism of inactivated X chromosomes (47, 48). Theoretically, epigenetic silencing of the X chromosome in the early stages of embryonic development is random, and the ratio of inactivated X chromosomes of maternal and paternal origin is 50:50. However, pathogenic mutations in X-linked genes can cause skewing of X-inactivation, leading to disproportionality in the distribution of X chromosomes with mutant alleles (49). The presence of RP symptoms, but not respiratory symptoms in the heterozygous mother, can be explained by two hypotheses. The first one assumes skewing of X chromosome inactivation in the respiratory cells of females as the result of preferential selection of wild-type gene alleles in respiratory cells. Another hypothesis proposes that a small amount of normal RPGR protein is sufficient for the function of respiratory cilia (3).

## 4 Conclusion

This case report contributes to the assumption that *RPGR* mutations disrupt the function of cilia in multiple tissues, including the retina and respiratory tract, highlighting the essential role of RPGR protein in ciliary biology. Understanding the underlying molecular mechanisms can help in the development of targeted therapies and also improve therapeutic approaches and genetic counselling. Moreover, the identification of *RPGR* mutations in patients with RP and PCD and described genotype–phenotype correlations have important implications for clinical practice to recognise a broader spectrum of PCD phenotypes as well as for future research focused on the genetic basis of PCD, gene interactions, the pathways implicated in PCD pathogenesis, and the role of RPGR protein for the proper functioning of cilia in various tissues throughout the body.

## Data Availability

The original contributions presented in the study are included in the article/Supplementary Material, further inquiries can be directed to the corresponding author.
